# Propensity matched comparison of TAVI and SAVR in intermediate-risk patients with severe aortic stenosis and moderate-to-severe chronic kidney disease: a subgroup analysis from the German Aortic Valve Registry

**DOI:** 10.1007/s00392-022-02083-2

**Published:** 2022-09-08

**Authors:** Silvia Mas-Peiro, Gloria Faerber, Dimitra Bon, Eva Herrmann, Timm Bauer, Sabine Bleiziffer, Raffi Bekeredjian, Andreas Böning, Christian Frerker, Andreas Beckmann, Helge Möllmann, Stephan Ensminger, Christian W. Hamm, Friedhelm Beyersdorf, Stephan Fichtlscherer, Thomas Walther, For the GARY-Executive Board

**Affiliations:** 1 Department of Cardiology, University Hospital Frankfurt am Main, Theodor-Stern-Kai 7, 60590 Frankfurt am Main, Germany; 2 German Center for Cardiovascular Research, DZHK, Partner Site Rhine-Main, Rhine-Main, Germany; 3 Department of Cardiothoracic Surgery, Jena University Hospital, Friedrich-Schiller-University of Jena, Jena, Germany; 4 Institute of Biostatistics and Mathematical Modelling, University Hospital Frankfurt am Main, Frankfurt am Main, Germany; 5 Department of Cardiology, Sana Klinikum Offenbach, Offenbach, Germany; 6 Department of Cardiothoracic Surgery, Heart and Diabetes Center NRW, University Hospital of the Ruhr-University Bochum, Bad Oeynhausen, Germany; 7 Department of Cardiology, Robert-Bosch Hospital, Stuttgart, Germany; 8 Department of Cardiothoracic Surgery, University Hospital Giessen, Giessen, Germany; 9 Department of Internal Medicine III, Faculty of Medicine and University Hospital Cologne, University of Cologne, Cologne, Germany; 10 German Society of Thoracic and Cardiovascular Surgery, Langenbeck-Virchow-Haus, Berlin, Germany; 11 Department of Cardiology, St. Johannes Hospital, Dortmund, Germany; 12 Department of Cardiac and Thoracic Vascular Surgery, University Hospital Schleswig-Holstein, Lübeck, Germany; 13 Department of Cardiology, Kerckhoff Campus, University of Giessen, Giessen, Germany; 14 Department of Cardiovascular Surgery, University Heart Center Freiburg-Bad Krozingen, University Hospital Freiburg, Freiburg, Germany; 15 Medical Faculty of the Albert-Ludwigs-University Freiburg, Freiburg, Germany; 16 Department of Cardiothoracic Surgery, University Hospital Frankfurt am Main, Frankfurt am Main, Germany

**Keywords:** Transcatheter aortic valve implantation, Surgical aortic valve replacement, Aortic stenosis, Chronic kidney disease, Mortality

## Abstract

**Objective:**

We compared TAVI vs. SAVR in patients with moderate-to-severe chronic kidney disease (eGFR 15–60 ml/min/1.73 m^2^) for whom both procedures could possibly be considered (age ≤ 80 years, STS-score 4–8).

**Background:**

According to both ACC/AHA and ESC/EACTS recent guidelines, aortic stenosis may be treated with either transcatheter (TAVI) or surgical (SAVR) aortic valve replacement in a subgroup of patients. A shared therapeutic decision is made by a heart team based on individual factors, including chronic kidney disease (CKD).

**Methods:**

Data from the large nationwide German Aortic Valve Registry were used. A propensity score method was used to select 704 TAVI and 374 SAVR matched patients. Primary endpoint was 1-year survival. Secondary endpoints were clinical complications, including pacemaker implantation, vascular complications, myocardial infarction, bleeding, and the need for new-onset dialysis.

**Results:**

One-year survival was similar (HR [95% CI] for TAVI 1.271 [0.795, 2.031], *p* = 0.316), with no divergence in Kaplan–Meier curves. In spite of post-procedural short-term survival being numerically higher for TAVI patients and 1-year survival being numerically higher for SAVR patients, such differences did not reach statistical significance (96.4% vs. 94.2%, *p* = 0.199, and 86.2% vs. 81.2%, *p* = 0.316, respectively). In weighted analyses, pacemaker implantation, vascular complications, and were significantly more common with TAVI; whereas myocardial infarction, bleeding requiring transfusion, and longer ICU-stay and overall hospitalization were higher with SAVR. Temporary dialysis was more common with SAVR (*p* < 0.0001); however, a probable need for chronic dialysis was rare and similar in both groups.

**Conclusion:**

Both TAVI and SAVR led to comparable and excellent results in patients with moderate-to-severe CKD in an intermediate-risk population of patients with symptomatic severe aortic stenosis for whom both therapies could possibly be considered.

**Supplementary Information:**

The online version contains supplementary material available at 10.1007/s00392-022-02083-2.

## Introduction

In the last decade, transcatheter aortic valve implantation (TAVI) has emerged as an alternative to conventional surgical aortic valve replacement (SAVR), initially in patients with a high or prohibitive surgical risk [1]. In recent years, indications for TAVI have been expanded to intermediate-risk patients [2, 3], and finally to low-risk patients [4–6]. According to recent European guidelines [7], in symptomatic severe aortic stenosis (AS), when both SAVR and TAVI are technically suitable, either SAVR or TAVI can be recommended, “according to individual clinical, anatomical and procedural characteristics” as evaluated by the heart team and after a discussion with the patient, in those patients younger than 75 years with intermediate surgical risk (4–8 score in STS-PROM/EuroScore II). According to current American guidelines [8], in the absence of contraindications, either SAVR or TAVI are also recommended “after shared decision-making about the balance between expected patient longevity and valve durability” in patients 65–80 years old not having a high surgical risk (particularly, STS-PROM/EuroScore II < 8). Thus, in a subgroup of symptomatic patients the decision to use TAVI versus SAVR is recommended to be based on a multidisciplinary heart team case-to-case discussion [7] and/or a shared process [8] to take into account patient´s factors affecting quality of life, as well as patient’s motivation. Therefore, despite slight differences in recommended age limits (65–80 years and < 75 years in American and European guidelines, respectively), both TAVI and SAVR can possibly be considered in a subgroup of patients according to international guidelines.

In order to facilitate patient selection for such therapies, previous studies have identified a number of patient factors and biomarkers having a potential impact on prognosis after SAVR or TAVI. Among them, special attention has been paid to renal dysfunction markers [9]. CKD has been shown to have a substantial impact on prognosis, with significant risk thresholds being different for TAVI or SAVR [10]. Although several studies [11–14] and meta-analyses [15, 16] have compared TAVI vs. SAVR in patients with CKD using matching methods, to our knowledge, no specific analyses have been published for this specific subgroup of patients in which both therapies may be considered, using recently defined CKD risk thresholds for TAVI and SAVR [10].

Using a propensity-score matching of data from the large German Aortic Valve Registry (GARY), we compared the clinical results of TAVI vs. SAVR in patients with moderate-to-severe CKD (eGFR 15–60 ml/min/1.73 m^2^) for whom both TAVI and SAVR could possibly be considered.

## Methods

### The German Aortic Valve Registry (GARY)

GARY design and protocol have been previously reported [17]. From January 2011 to December 2015, all consecutive patients undergoing TAVI or SAVR in the vast majority of German hospitals performing such procedures (*n* = 88) were enrolled. Only patients who refused to participate were excluded. The study was approved by the institutional review board/ethics committee of all participating sites and written informed consent was obtained from all patients.

### Study population

Data were provided by the independent BQS institute, and all registry patients with severe AS undergoing TAVI or SAVR and having 1-year follow-up were considered. Exclusion criteria were the same used in previous GARY reports on CKD and have been previously published [10], the main ones being missing/outlier data for age, creatinine levels or simultaneous use of additional surgical procedures.

To study a subcohort of patients for whom both TAVI and SAVR could possibly be considered, a subpopulation based on age (65–80 years) and overall clinical risk status (STS-score, 4–8) was selected. Such patients fulfilled the criteria for both TAVI and SAVR according to American guidelines. Our upper age limit was higher than the recently suggested in European guidelines (75 years) to make our population more inclusive at a global level. Nevertheless, we also performed a whole alternative analysis for patients between 65 and 75 years, to reflect patients selected according to European guidelines.

**Fig. 1:**
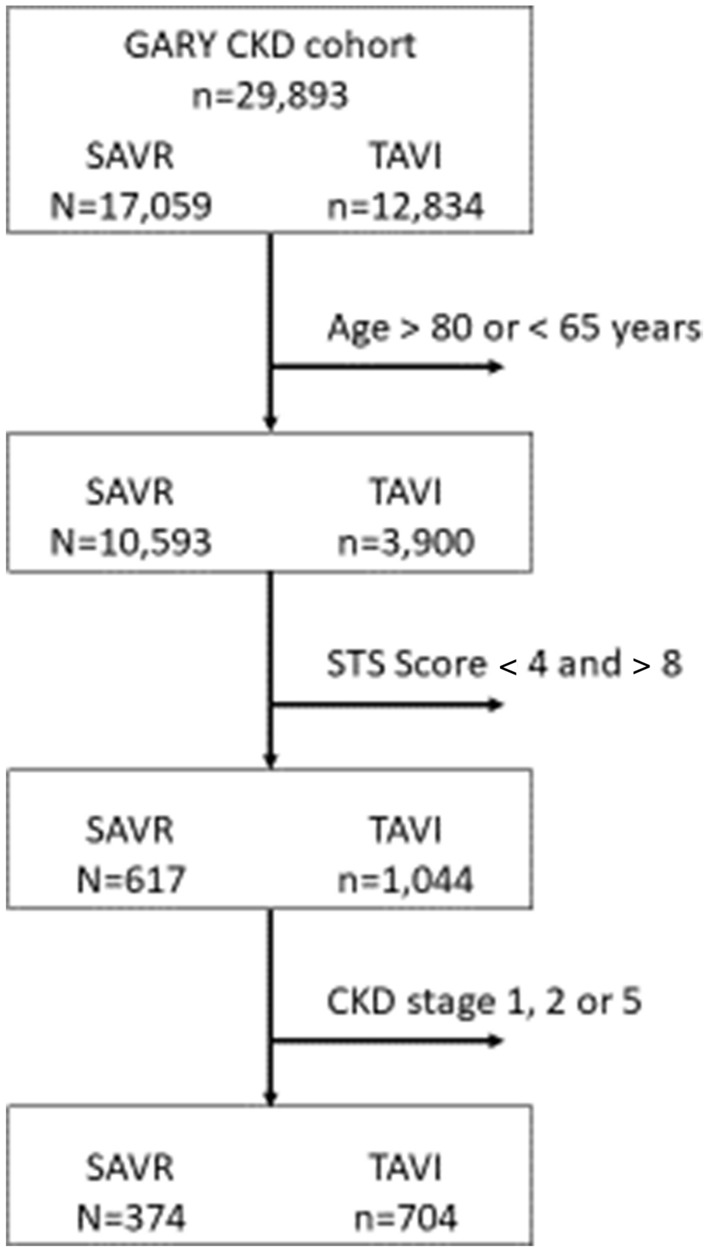
Flowchart of patient selection

CKD stages and substages were defined using estimated glomerular filtration rate (eGFR) (ml/min/1.73 m^2^) values based on Modification of Diet in Renal Disease (MDRD) [18] as suggested for cardiovascular diseases [19]. It has recently been shown that eGFR is significantly correlated with survival. CKD stage ≥ 3a was a significant independent risk factor for SAVR, and CKD stage ≥ 3b was a risk factor for TAVI [10]. Based on those findings, for the present study we selected patients having CKD stages 3a, 3b, and 4. Patients with very severe CKD (stage 5) were excluded from present analyses and the results of TAVI and SAVR in patients on dialysis in the registry have been published separately [20]. Dialysis may result in specific complications that can have a unique impact on clinical outcomes after the procedure. Moreover, excluding such patients allows our results to be contrasted with the results of a number of previous studies on the topic in which CKD 5 patients were also excluded [21, 22].The study flowchart is shown in Fig. 1.

### Outcomes

One-year survival was the primary endpoint. Causes of death and complications were also assessed. Specifically, cardiac (myocardial infarction, new-onset of atrial fibrillation, permanent pacemaker implantation) and other vascular complications (including stroke, and transfusion needs) were carefully evaluated. Length of stay in intensive care unit (ICU) and in hospital were also assessed. Potential postprocedural acute kidney injury (AKI) was estimated based on new-onset of temporary or long-term dialysis.

### Statistical analysis

Statistical analyses were performed with R software (R Foundation for Statistical Computing, Vienna, Austria). A significance level of *α* = 0.05 was used in all statistical tests. Categorical data are shown as frequencies and percentages. Mean ± SE values are used to present continuous data after propensity score weighting.

In the selected subcohort, a propensity score analysis with matching weights (weighting to estimate the average treatment effect of the TAVI-treated group) was performed. Variables were included in the model based on those univariately predicting mortality in TAVI and SAVR patients in the overall analysis of kidney dysfunction impact in GARY registry plus those considered to be clinically relevant: age, gender, STS-score, New York Heart Association (NYHA) class III/IV, atrial fibrillation, mitral regurgitation ≥ 2°, previous cardiac surgery, left ventricular ejection fraction (LVEF), pulmonary hypertension, and insulin-dependent diabetes. Propensity scores were estimated by boosted logistic regression methods using the twang package of R. Unweighted and weighted comparisons between the groups included logistic and linear regression and weighted Cox regression. Since a statistical difference remained between groups for two variables even after the adjustment procedure, an additional adjustment was performed by means of a Cox regression. Weighted Kaplan–Meier curves for 1-year cumulative survival were created for TAVI vs. SAVR.

## Results

**Fig. 2:**
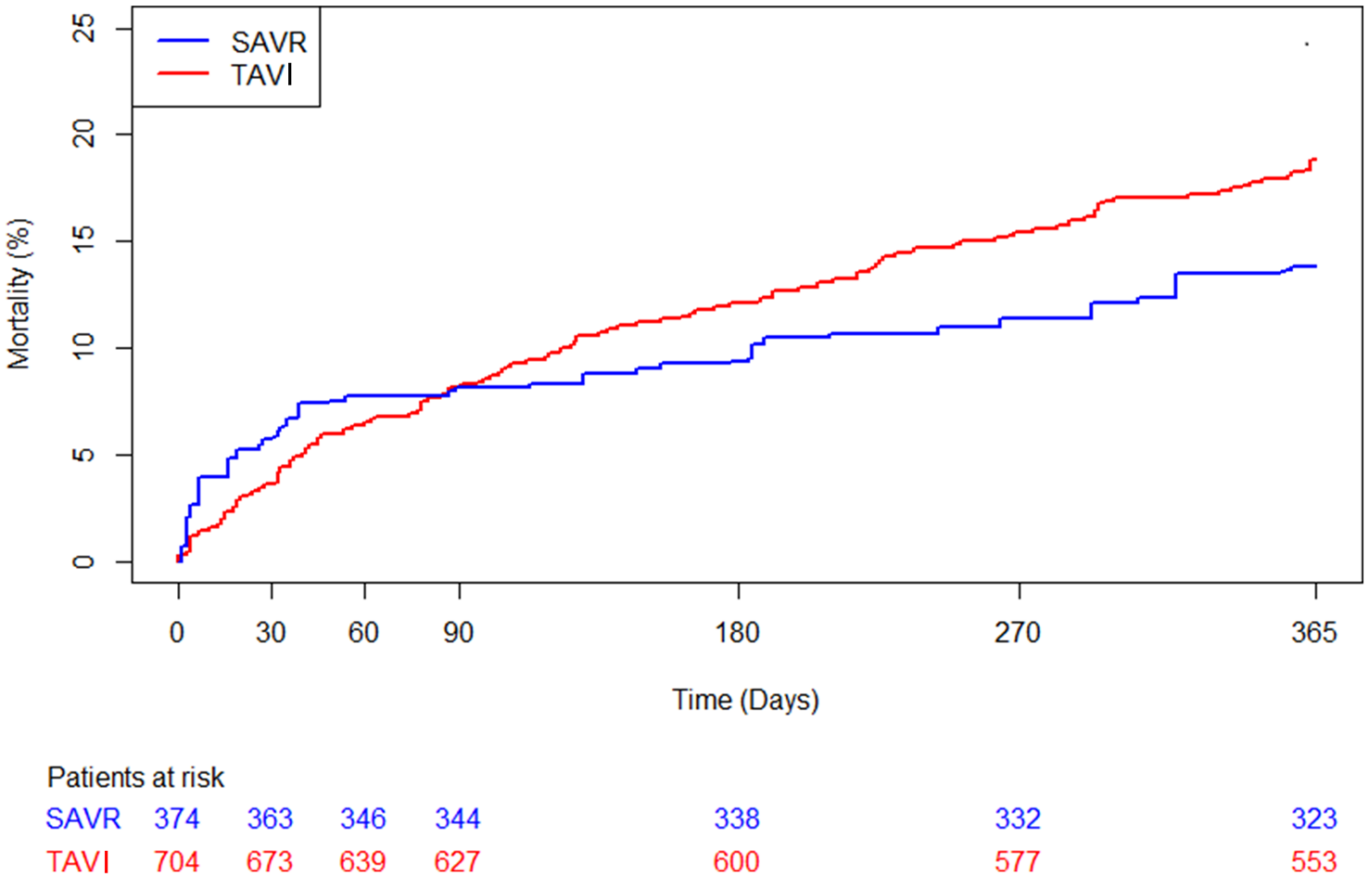
Kaplan–Meier curves for 1-year cumulative all-cause mortality in 704 TAVI and 374 SAVR patients after propensity score weighting (*p* = 0.316)

A total of 29,893 patients from the registry with 1-year follow-up were included: 12,834 were treated with TAVI and 17,059 underwent SAVR. A subgroup of 1078 patients would have been candidates to either TAVI or SAVR according to the aforementioned study criteria: 374 of them underwent SAVR and 704 were treated with TAVI. Data from these patients were used for a propensity matched comparison and only the results for this population are presented here. Mean age of the study population was 76 years and median STS-score was 5.3. Further comparisons of clinical characteristics for unadjusted and propensity score adjusted analysis are shown in Table 1. Adjustment for atrial fibrillation and pulmonary hypertension remained imperfect; therefore, Cox regression for 1-year survival also includes both variables for further adjustment (Table 2). The Cox regression model for one-year survival did not show significant differences between TAVI and SAVR, with HR (95% CI) for TAVI being 1.271 (0.795, 2.031), *p* = 0.316; and no significant divergence was found in Kaplan–Meier curves (see Fig. 2). In spite of post-procedural short-term (30-day) survival being numerically higher for TAVI patients and 1-year survival being numerically higher for SAVR patients, such differences did not reach statistical significance (96.4% vs. 94.2%, *p* = 0.199, and 86.2% vs. 81.2%, *p* = 0.316, respectively).

**Table 1:** Demographic and clinical characteristics in patients before and after propensity score analysis

	SAVR unweighted (*n* = 374)	SAVR weighted (*n* = 374)	TAVI (*n* = 704)	*p* unweighted	*p* weighted
Age (years)	75.3 (0.2)	76.4 (0.1)	76.7 (0.1)	< 0.0001	0.1924
Age classes					
< 70	45 (12.0%)	(4.3%)	24 (3.4%)	< 0.0001	0.4101
70–74	99 (26.5%)	(20.4%)	130 (18.5%)	0.0023	0.3913
75–79	230 (61.5%)	(75.3%)	550 (78.1%)	< 0.0001	0.2393
Gender (female) (%)	257 (68.7%)	(59.8%)	408 (58.0%)	0.0006	0.5185
BMI (kg/m^2^)	30.517 (0.326)	29.098 (0.283)	29.631 (0.237)	0.0282	0.1552
BMI classes					
< 18.5 kg/m^2^	2 (0.5%)	(0.6%)	8 (1.2%)	0.3445	0.3644
18.5–25.0 kg/m^2^	79 (21.6%)	(25.7%)	171 (24.6%)	0.2755	0.6727
≥ 25.0 kg/m^2^	284 (77.8%)	(73.7%)	515 (74.2%)	0.1961	0.8291
Creatinine (mg/dl)	1.484 (0.025)	1.496 (0.023)	1.497 (0.018)	0.6863	0.9799
Estimated GFR (eGFR) (ml/min/1.73 m^2^)	41.494 (0.583)	41.690 (0.520)	41.397 (0.425)	0.8926	0.6714
CKD in groups					
Stage 3a	169 (45.2%)	(45.9%)	284 (40.3%)	0.1251	0.0522
Stage 3b	133 (35.6%)	(37.5%)	299 (42.5%)	0.0277	0.0782
Stage 4	72 (19.3%)	(16.6%)	121 (17.2%)	0.4004	0.7973
NYHA					
I	2 (0.5%)	(0.2%)	2 (0.3%)	0.5262	0.8463
II	79 (21.1%)	(14.2%)	83 (11.8%)	< 0.0001	0.2042
III	254 (67.9%)	(72.2%)	529 (75.1%)	0.0115	0.2435
IV	39 (10.4%)	(13.3%)	90 (12.8%)	0.2573	0.7728
NYHA (III/IV)	293 (78.3%)	(85.5%)	619 (87.9%)	< 0.0001	0.2188
Previous MI (%)	28 (7.5%)	(14.1%)	102 (14.6%)	0.0009	0.8357
Permanent pacemaker (%)	33 (8.8%)	(16.2%)	96 (14.2%)	0.0122	0.3411
Atrial fibrillation (%)	87 (23.3%)	(28.3%)	270 (38.4%)	< 0.0001	0.0002
Previous cardiac surgery (%)	52 (13.9%)	(30.4%)	222 (31.6%)	< 0.0001	0.6713
EF (%)	57.7 (0.8)	52.3 (0.7)	50.9 (0.5)	< 0.0001	0.1445
EF ≤ 30% (%)	18 (5.6%)	(12.7%)	90 (13.5%)	0.0003	0.6961
Hypertension (%)	347 (92.8%)	(91.6%)	654 (93.6%)	0.6261	0.1859
Neurological dysfunction (%)	46 (12.3%)	(16.7%)	96 (13.6%)	0.5369	0.1313
Lung disease (%)	133 (35.6%)	(32.8%)	212 (30.1%)	0.0683	0.3114
Pulmonary hypertension > 55 mmHg (%)	30 (8.1%)	(14.3%)	137 (19.8%)	< 0.0001	0.0128
Diabetes (%)	247 (66.0%)	(61.3%)	434 (61.6%)	0.1547	0.8894
Insulin dependent diabetes (%)	156 (63.2%)	(59.6%)	242 (55.8%)	0.0600	0.2958
AKL-Score	3.23 (0.12)	4.05 (0.12)	4.45 (0.09)	< 0.0001	0.0143
Euro-Score	12.4 (0.6)	17.8 (0.6)	19.1 (0.4)	< 0.0001	0.1079
STS-Score	5.11 (0.05)	5.35 (0.05)	5.40 (0.04)	< 0.0001	0.4552

Percentages and estimated weighted percentages for categorical variables and means with standard errors or weighted means with standard errors for quantitative variables are shown

*BMI* body mass index, *COPD* chronic obstructive pulmonary disease, *CKD* chronic kidney disease, *EF* ejection fraction, *GFR* glomerular filtration rate, *MI* myocardial infarction, *NYHA* New York Heart Association, *SAVR* surgical aortic valve replacement, *TAVI* transcatheter aortic valve implantation

**Table 2:** Hazard ratios (HR) from adjusted Cox regression for 1-year survival using weights from the propensity score analysis

	HR (95% CI)	*p*-value
Univariate analysis		
TAVI vs. SAVR	1.368 (0.860, 2.178)	0.1860
Multivariate analysis		
TAVI vs. SAVR	1.235 (0.756, 2.018)	0.3164
Atrial fibrillation (yes)	1.368 (0.927, 2.020)	0.1015
Pulmonary hypertension > 55 mmHg	1.740 (1.020, 2.967)	0.0318

As adjustment was less optimal for atrial fibrillation and pulmonary hypertension, these variables are also included in a multivariate analysis

*SAVR* surgical aortic valve replacement, *TAVI* transcatheter aortic valve implantation

Procedural and post-procedural complications without and with adjustment in this subpopulation (*n* = 1078) are shown in Table 3. In weighted analyses, permanent pacemaker implantation, and vascular complications were significantly more common in TAVI patients, whereas SAVR-treated patients had significantly higher rates of myocardial infarction, bleeding requiring transfusion and a higher length of ICU-stay and overall hospital stay. At hospital discharge, treatment with antiplatelet drugs was significantly more common in TAVI patients (89.9% vs. 55.3%, *p* < 0.0001) and treatment with anticoagulants was significantly more common in SAVR patients (70.5% vs. 49.5%, *p* < 0.0001). The use of temporary dialysis and/or the probable need for long-term dialysis were based on clinician’s assessment at hospital discharge. New onset of temporary dialysis was significantly higher in SAVR patients (10.7% vs. 3.3%; *p* < 0.0001). However, probable need for chronic dialysis was rare and similar with both therapies. Further complications at 1 year are shown in Supplementary Table 1.

**Table 3:** Procedural results and post-procedural complications with and without propensity score weighted adjustment

	SAVR unweighted (*n* = 374)	SAVR weighted (*n* = 374)	TAVI (*n* = 704)	*p* unweighted	*p* weighted
Procedural results					
Urgent *n* (%)	77 (20.6%)	(19.0%)	87 (12.4%)	0.0004	0.0014
Procedure duration (min) mean (SE)	170.0 (2.1)	176.0 (2.2)	90.5 (1.8)	< 0.0001	< 0.0001
Pericardial tamponade *n* (%)	0 (0.0%)	(0.0%)	2 (0.3%)	0.9947	0.9943
Requested by patient *n* (%)	0 (0.0%)	(0.0%)	185 (26.3%)	0.9734	0.9717
Vascular complication *n* (%)	1 (0.3%)	(0.3%)	27 (3.8%)	0.0081	0.0018
Post implant mean gradient					
< 10 mmHg *n* (%)	65 (34.8%)	(40.5%)	272 (60.6%)	< 0.0001	< 0.0001
10–14 mmHg *n* (%)	50 (26.7%)	(25.8%)	111 (24.7%)	0.5942	0.7566
≥ 15 mmHg *n* (%)	72 (38.5%)	(33.7%)	66 (14.7%)	< 0.0001	< 0.0001
Diameter, mean (SE)	22.4 (0.1)	22.5 (0.1)	26.4 (0.1)	< 0.0001	< 0.0001
Post-procedural data					
Stroke *n* (%)	4 (1.1%)	(0.8%)	8 (1.1%)	0.9360	0.5701
TIA *n* (%)	5 (1.4%)	(1.2%)	2 (0.3%)	0.0630	0.0848
Myocardial infarction *n* (%)	5 (1.3%)	(2.8%)	1 (0.1%)	0.0400	0.0038
New-onset atrial fibrillation *n* (%)	81 (22.0%)	(25.1%)	192 (28.0%)	0.0351	0.2618
New-onset pacemaker *n* (%)	15 (5.7%)	(8.2%)	48 (13.3%)	0.0025	0.0331
Bleeding ≥ 2 RBC units *n* (%)	207 (90.0%)	(91.9%)	141 (82.0%)	0.0212	0.0013
Vascular complication *n* (%)	2 (0.5%)	(0.6%)	47 (6.7%)	0.0004	< 0.0001
Antiplatelet drug *n* (%)	222 (59.7%)	(55.3%)	631 (89.9%)	< 0.0001	< 0.0001
Anticoagulant *n* (%)	252 (67.9%)	(70.5%)	346 (49.5%)	< 0.0001	< 0.0001
New-onset dialysis					
Probably temporary *n* (%)	37 (9.9%)	(10.7%)	23 (3.3%)	< 0.0001	< 0.0001
Probably chronic *n* (%)	2 (0.5%)	(0.1%)	2 (0.3%)	0.5262	0.4600
Post-OP stay in ICU (days), mean (SE)	4.7 (0.3)	5.0 (0.3)	3.3 (0.2)	0.0002	< 0.0001

Percentages and estimated weighted percentages for categorical variables and means with standard errors or weighted means with standard errors (ES) for quantitative variables are shown

*CPB* cardiopulmonary bypass, *HLM* heart–lung machine, *ICU* intermediate care unit, *RBC* red blood cell, *OP* operation, *SAVR* surgical aortic valve replacement, *TAVI* transcatheter aortic valve implantation, *TIA* transient ischemic attack

The results of the alternative analysis for the population aged 65–75 years (i.e., according to European guidelines age limits) are shown in Supplementary Fig. 1 and Supplementary Tables 2, 3, 4. All survival results were very similar. Complications rates were also numerically similar, although significance was not achieved for some of them in this much smaller patient cohort (Supplementary Table 5).

## Discussion

Matched comparisons [11–14] of the impact of CKD on clinical outcomes after TAVI vs. SAVR in patients with severe AS have shown mixed results, but two meta-analyses have suggested overall results to be favourable to TAVI [15, 16]. However, to our knowledge, this is the first study with selection criteria based on recently defined CKD risk thresholds for TAVI and SAVR [10] in which clinical outcomes after TAVI vs. SAVR have been compared using a propensity score adjustment method in patients in which both TAVI and SAVR could possibly be considered according to recent studies. To avoid further artificial subgroup analysis, we used a propensity score weighting method [23, 24].

In our population of patients with moderate-to-severe CKD, TAVI and SAVR therapies showed essentially similar survival results at 1-year follow-up. Thus, our results based on real-world data from a large representative European registry in this subpopulation confirm the appropriateness of recent international recommendations. However, some differences were found regarding the most common complications between both therapies. Specifically, significant bleeding, and myocardial infarction were more common with SAVR, whereas pacemaker need was more common with TAVI. As expected, vascular complications were more common with TAVI and hospital stay was longer with SAVR. These differences in complication rates may be of interest when discussing the options with the patient and to inform the future selection of therapeutic approaches by heart teams when considering specific comorbidities and risks.

Acute kidney injury (AKI) is a matter of concern after SAVR and also after TAVI since the introduction of transcatheter procedures [25]. Some large studies [13, 26, 27] and meta-analyses [28–30] have shown a lower risk of postprocedural AKI with TAVI. In our analysis, although a significantly higher need for acute newly onset dialysis with SAVR was confirmed, the need for long-term dialysis was very low and similar with TAVI and SAVR.

Apart from the specific recommendations for patients with particular comorbidities or specific anatomic or procedural characteristics, recent 2021 ESC guidelines suggest a decision on TAVI vs. SAVR by a heart team, based on individual factors and after a discussion with the patient, in those patients younger than 75 years with intermediate surgical risk (4–8 score in STS-PROM/EuroScore II) [7]. Current 2020 ACC/AHA guidelines also provide some specific orientation for the low and intermediate risk (STS ≤ 8) group and suggest considering either SAVR or TAVI in 65-to-80-years-old symptomatic patients after shared decision-making, which requires taking into account expected longevity, valve durability, and individual factors [8].

The impact of CKD on TAVI and SAVR results has been widely recognized and studied. Leaving aside patients with very severe CKD (stage 5) that were not included in our analysis, most real-world data suggest that mild (stage 2) CKD has no prognostic effect, whereas moderate-to-severe CKD (stages 3–4) is a consistent risk factor [10, 11, 31–33]. Previous direct comparisons of TAVI vs. SAVR in patients with more-than-mild CKD are scarce and usually include all treated patients, not just those eligible for both therapies according to guidelines. A meta-analysis by Cheng et al. suggested TAVI could be better than SAVR in CKD patients [15]; however, 4 out of 10 studies exclusively included patients on dialysis, and other 4 studies included patients with end-stage renal disease or CKD 5 stage. Similar results have been found in another meta-analysis [16]. D’Errigo et al. reported slightly better results with SAVR, not reaching significance, in 170 propensity score matched pairs of patients in CKD 3b-5 stages from the Italian OBSERVANT registry [11]. On the contrary, Shavit et al., in a small not matched study in mostly very old patients [22], and Kumar et al. in a propensity score-matched study of all-age and all-CKD level patients [34], found better results with TAVI. However, these studies were previous to current guidelines and did not select patients eligible for both TAVI and SAVR based on current age and surgical risk criteria.

The main strengths of our study include the use of a large and representative real-world database at a national level, the selection of patients essentially based on most recent international guidelines; and the statistical meaningful comparison of TAVI and SAVR using modern propensity matched analyses. Moreover, since American and European guidelines are not fully consistent when defining patients who are candidates to TAVI and SAVR, we managed to select a population for which recent American guidelines recommend either TAVI or SAVR, and confirmed the same results in an equivalent population based on European guidelines. Some limitations must also be acknowledged. First, the initial cohorts were not comparable in terms of clinical parameters and a detailed propensity matching procedure was needed to allow meaningful comparisons. As in any observational registry study, even with a very large sample and a prospective follow-up as used in GARY, some unidentified confounding factors could remain in spite of the thorough adjustment, and the results must be interpreted cautiously. Specific randomized controlled trials will be needed to definitely address this important issue, and some ongoing trials are currently comparing SAVR vs TAVI in low/intermediary risk patients (e.g., DEDICATE study [35]). However, properly adjusted analyses from large registries do have a role. They can provide us with some insights on the topic when making therapeutic decisions on our current patients, while waiting for clinical trials’ results. Furthermore, ongoing trials are not specifically focused on our target population (intermediate risk plus moderate-to-severe CKD); conclusions based on trial’s sub-analyses will be highly informative, but will also require caution in interpretation because they do not reflect a truly randomized comparison. Both American and European guidelines have been recently issued; it will take some time to have trials results available for such specific cohorts that are of particular interest for clinicians. Moreover, real-world data have an additional complementary role. Even though clinical trials are the only way to prove an effect, they include highly selected patients that are carefully controlled and are not fully representative for the real patients being treated in daily clinical practice. Our observational results, based on data from the vast majority of procedures performed at a national level, do reflect the real world results in patients undergoing either TAVI or SAVR. Second, our data do not include a substantial proportion of the most recent prosthetic valve devices; however, at the moment there are no reasons to expect different results in CKD patients with the latest devices. Third, using estimated glomerular filtration rates based on creatinine levels is also a limitation. Fourth, our findings are based on short- and mid-term (1-year mortality) outcomes and do not necessarily reflect longer-term results. Fifth, our conclusions are not applicable to CKD 5 patients frequently receiving dialysis, because such patients were excluded from analyses. GARY results for patients on dialysis have been reported separately [20]. And sixth, complications were not reported according to current BARC and VARC criteria, as currently recommended, because such criteria were not yet in common use when GARY registry was started. Despite not using such categorization, our description of complications appears to be, however, fully illustrative.

In conclusion, we performed a propensity score-matched study based on a large nation-wide registry (GARY) in a selected subcohort of intermediate-risk patients for whom both TAVI and SAVR could possibly be considered. Our results confirm that good and similar results are obtained with both therapies in patients with moderate-to-severe CKD and intermediate-risk. These findings can be considered by heart teams and will provide an additional tool for personalized decision-making in severe AS.

## Supplementary Information

Below is the link to the electronic supplementary material.

Supplementary file1 (TIF 11 KB)

Supplementary file2 (DOCX 43 KB)
