# Supplementary material for: Propensity matched comparison of TAVI and SAVR in intermediate-risk patients with severe aortic stenosis and moderate-to-severe chronic kidney disease: a subgroup analysis from the German Aortic Valve Registry
Source: Clin Res Cardiol. 2022 Sep 8;111(12):1387–95. doi: 10.1007/s00392-022-02083-2 (PMC9681690; doi:10.1007/s00392-022-02083-2)
Supplement: Supplementary file 2 — Supplementary file2 (DOCX 43 KB) [file 392_2022_2083_MOESM2_ESM.docx]

**Supplementary Table 1: Complications at 1 year after propensity score weighted adjustment.**

|  | **SAVR unweighted (n=374)** | **SAVR  (n=374)** | **TAVI  (n=704)** | **p unweighted** | **p weighted** |
| --- | --- | --- | --- | --- | --- |
| **Myocardial infarction** |  |  |  | 0.4154 | 0.0197 |
| yes n/N (%) | 5 (1.8 %) | (3.6 %) | 5 (1.1 %) |  |  |
| **New Pacemaker/ICD** |  |  |  | <0.0001 | 0.0009 |
| yes n/N (%) | 19 (7.5 %) | (10.0 %) | 73 (19.3 %) |  |  |
| **Stroke** |  |  |  | 0.1227 | 0.0452 |
| yes n/N (%) | 2 (0.7 %) | (0.5 %) | 11 (2.4 %) |  |  |
| **TIA** |  |  |  | 0.8615 | 0.4650 |
| yes n/N (%) | 272 (98.6 %) | (99.2 %) | 457 (98.7 %) |  |  |
| **Bypass** |  |  |  | 0.9951 | 0.9949 |
| yes n/N (%) | 0 (0.0 %) | (0.0 %) | 3 (0.6 %) |  |  |
| **PCI/Balloon Dilatation** |  |  |  | 0.6213 | 0.9435 |
| yes n/N (%) | 3 (1.1 %) | (1.4 %) | 7 (1.5 %) |  |  |
| **Further Hospitalization** |  |  |  | 0.0040 | 0.0014 |
| yes n/N (%) | 104 (37.1 %) | (37.0 %) | 224 (48.0 %) |  |  |
| **Further Hospitalization due to complications related to the aortic valve intervention** |  |  |  | 0.3422 | 0.0320 |
| yes n/N (%) | 16 (15.4 %) | (19.9 %) | 26 (11.6 %) |  |  |
| **Further Hospitalization due to heart or circulatory problems** |  |  |  | 0.7155 | 0.6822 |
| yes n/N (%) | 45 (44.1 %) | (44.2 %) | 94 (42.0 %) |  |  |
| **NYHA** |  |  |  |  |  |
| I n/N (%) | 106 (38.5 %) | (37.7 %) | 129 (27.8 %) | 0.0025 | 0.0023 |
| II n/N (%) | 95 (34.5 %) | (36.7 %) | 167 (36.0 %) | 0.6913 | 0.8284 |
| III n/N (%) | 65 (23.6 %) | (22.4 %) | 154 (33.2 %) | 0.0063 | 0.0006 |
| IV n/N (%) | 9 (3.3 %) | (3.2 %) | 14 (3.0 %) | 0.8467 | 0.8844 |
| **Comparison of current general health status to health status before intervention** |  |  |  |  |  |
| better n/N (%) | 168 (60.6 %) | (66.6 %) | 299 (64.6 %) | 0.2839 | 0.5328 |
| same as before n/N (%) | 67 (24.2 %) | (19.1 %) | 100 (21.6 %) | 0.4150 | 0.3729 |
| worse n/N (%) | 42 (15.2 %) | (14.3 %) | 64 (13.8 %) | 0.6148 | 0.8555 |
| **Patient satisfaction with the intervention** |  |  |  |  |  |
| very good n/N (%) | 123 (44.4 %) | (45.0 %) | 226 (49.7 %) | 0.1668 | 0.1782 |
| good n/N (%) | 114 (41.2 %) | (39.9 %) | 168 (36.9 %) | 0.2540 | 0.3729 |
| satisfactory n/N (%) | 25 (9.0 %) | (9.4 %) | 45 (9.9 %) | 0.6997 | 0.7979 |
| not satisfactory n/N (%) | 15 (5.4 %) | (5.7 %) | 16 (3.5 %) | 0.2195 | 0.1328 |

ICD: implantable cardioverter defibrillator; NYHA: New York Heart Association; PCI: percutaneous coronary intervention; SAVR: surgical aortic valve replacement; TAVI: transcatheter aortic valve replacement; TIA: transient ischemic attack

**Supplementary Table 2.** Demographic and clinical characteristics in patients before and after propensity score analysis. Percentages and estimated weighted percentages for categorical variables and means with standard errors or weighted means with standard errors for quantitative variables are shown. Only patients with age between 65 and 75 years are included.

|  | **SAVR unweighted (n=181)** | **SAVR weighted**  **(n=181)** | **TAVI**  **(n=222)** | **p unweighted** | **p weighted** |
| --- | --- | --- | --- | --- | --- |
| Age (years) | 72.2 (0.2) | 73.2 (0.2) | 73.3 (0.2) | 0.0002 | 0.6293 |
| Age < 70 years | 45 (24.9 %) | (14.9 %) | 24 (10.8 %) | 0.0003 | 0.2228 |
| Gender (Female) (%) | 127 (70.2 %) | (51.7 %) | 107 (48.2 %) | <0.0001 | 0.4876 |
| BMI (kg/m²) | 31.6 (0.5) | 29.8 (0.5) | 30.1 (0.5) | 0.0316 | 0.6146 |
| BMI classes |  |  |  |  |  |
| < 18.5 kg/m² | 1 (0.6 %) | (0.5 %) | 5 (2.3 %) | 0.2071 | 0.2049 |
| 18.5 to 25.0 kg/m² | 36 (20.7 %) | (24.2 %) | 45 (20.4 %) | 0.9362 | 0.3640 |
| ≥ 25.0 kg/m² | 137 (78.7 %) | (75.2 %) | 171 (77.4 %) | 0.7461 | 0.6244 |
| Creatinine | 1.57 (0.04) | 1.69 (0.05) | 1.58 (0.04) | 0.8576 | 0.0635 |
| GFR | 40.3 (0.9) | 39.4 (0.9) | 41.6 (0.8) | 0.2835 | 0.0783 |
| CKD in Groups |  |  |  |  |  |
| Stage 3a | 75 (41.4 %) | (40.8 %) | 95 (42.8 %) | 0.7839 | 0.6895 |
| Stage 3b | 62 (34.3 %) | (32.0 %) | 85 (38.3 %) | 0.4029 | 0.1945 |
| Stage 4 | 44 (24.3 %) | (27.2 %) | 42 (18.9 %) | 0.1899 | 0.0511 |
| NYHA |  |  |  |  |  |
| I | 1 (0.6 %) | (0.2 %) | 0 (0.0 %) | 0.9957 | 0.9984 |
| II | 31 (17.1 %) | (12.3 %) | 32 (14.4 %) | 0.4562 | 0.5367 |
| III | 131 (72.4 %) | (75.4 %) | 159 (71.6 %) | 0.8669 | 0.3946 |
| IV | 18 (9.9 %) | (12.1 %) | 31 (14.0 %) | 0.2214 | 0.5935 |
| NYHA (III/IV) | 149 (82.3 %) | (87.6 %) | 190 (85.6 %) | 0.3731 | 0.5664 |
| Previous MI (%) | 19 (10.5 %) | (25.5 %) | 42 (19.1 %) | 0.0186 | 0.1278 |
| Permanent pacemaker (%) | 16 (8.8 %) | (18.4 %) | 27 (12.9 %) | 0.2021 | 0.1397 |
| Atrial fibrillation (%) | 44 (24.3 %) | (23.3 %) | 77 (34.7 %) | 0.0244 | 0.0150 |
| Previous cardiac surgery (%) | 32 (17.8 %) | (42.5 %) | 94 (42.3 %) | <0.0001 | 0.9824 |
| EF (%) | 58.1 (1.1) | 50.2 (1.2) | 48.7 (1.0) | <0.0001 | 0.3470 |
| EF ≤30% (%) | 8 (5.0 %) | (15.3 %) | 37 (17.7 %) | 0.0005 | 0.5398 |
| Hypertension (%) | 168 (92.8 %) | (88.3 %) | 204 (93.2 %) | 0.8966 | 0.0969 |
| Neurological dysfunction (%) | 28 (15.5 %) | (25.6 %) | 33 (14.9 %) | 0.8662 | 0.0083 |
| Lung disease (%) | 69 (38.1 %) | (32.8 %) | 81 (36.5 %) | 0.7356 | 0.4469 |
| Pulmonary hypertension >55 mmHg (%) | 14 (7.8 %) | (9.1 %) | 38 (17.4 %) | 0.0061 | 0.0207 |
| Diabetes (%) | 126 (69.6 %) | (59.2 %) | 149 (67.1 %) | 0.5925 | 0.1048 |
| Insulin dependent diabetes (%) | 88 (69.8 %) | (73.6 %) | 92 (61.7 %) | 0.1602 | 0.0515 |
| AKL-Score | 2.80 (0.18) | 4.02 (0.25) | 3.81 (0.16) | <0.0001 | 0.5129 |
| Euro-Score | 11.6 (0.9) | 18.7 (1.1) | 19.1 (0.8) | <0.0001 | 0.7699 |
| STS-Score | 5.14 (0.08) | 5.37 (0.079) | 5.36 (0.07) | 0.0342 | 0.8971 |

BMI: body mass index; COPD: chronic obstructive pulmonary disease; CKD: chronic kidney disease; EF: ejection fraction; GFR: glomerular filtration rate; MI: myocardial infarction; NYHA: New York Heart Association; SAVR: surgical aortic valve replacement; TAVI: transcatheter aortic valve replacement

**Supplementary Table 3.** Hazard ratios (HR) from adjusted Cox regression for 1-year survival using weights from the propensity score analysis. As adjustment was less optimal for atrial fibrillation and pulmonary hypertension, these variables are also included in a multivariate analysis. Only patients with age between 65 and 75 years are included.

| **Univariate Analysis** | **HR (95% CI)** | **p-value** |
| --- | --- | --- |
| TAVI vs. SAVR | 2.263 (0.972, 5.266) | 0.0581 |
| **Multivariate Analysis** | **HR (95% CI)** | **p-value** |
| TAVI vs. SAVR | 2.192 (0.963, 4.986) | 0.0614 |
| Atrial Fibrillation (yes) | 1.174 (0.679, 2.020) | 0.5665 |
| Pulmonary Hypertension > 55 mmHg | 0.892 (0.437, 1.821) | 0.7532 |

SAVR: surgical aortic valve replacement; TAVI: transcatheter aortic valve replacement**Supplementary** **Table 4**. Procedural results and post-procedural data with and without propensity score weighted adjustment. Percentages and estimated weighted percentages for categorical variables and means with standard errors or weighted means with standard errors (ES) for quantitative variables are shown. Only patients with age between 65 and 75 years are included.

|  | **SAVR unweighted (n=181)** | **SAVR weighted**  **(n=181)** | **TAVI**  **(n=222)** | **p unweighted** | **p weighted** |
| --- | --- | --- | --- | --- | --- |
| **Procedural results** |  |  |  |  |  |
| Urgent n (%) | 34 (18.8 %) | (18.7 %) | 27 (12.2 %) | 0.0668 | 0.0743 |
| Procedure Duration (min) mean (se) | 173.2 (3.0) | 183.9 (3.6) | 87.7 (3.1) | <0.0001 | <0.0001 |
| Pericardial tamponade n (%) | 0 (0.0 %) | (0.0 %) | 1 (0.5 %) | 0.9962 | 0.9965 |
| Requested by patient n (%) | 0 (0.0 %) | (0.0 %) | 60 (27.0 %) | 0.9815 | 0.9829 |
| Vascular complication n (%) | 1 (0.6 %) | (0.9 %) | 8 (3.6 %) | 0.0736 | 0.1150 |
| Post Implant Mean Gradient |  |  |  |  |  |
| <10mmHg n (%) | 30 (31.3 %) | (40.3 %) | 70 (51.5 %) | 0.0024 | 0.1143 |
| 10-14mmHg n (%) | 22 (22.9 %) | (25.7 %) | 39 (28.7 %) | 0.3272 | 0.6410 |
| ≥15mmHg n (%) | 44 (45.8 %) | (34.0 %) | 27 (19.9 %) | <0.0001 | 0.0224 |
| Diameter, mean (se) | 22.4 (0.2) | 22.8 (0.2) | 26.6 (0.1) | <0.0001 | <0.0001 |
|  |  |  |  |  |  |
| **Post-procedural complications** |  |  |  |  |  |
| Stroke n (%) | 2 (1.1 %) | (1.0 %) | 4 (1.8 %) | 0.5718 | 0.5294 |
| TIA n (%) | 2 (1.1 %) | (1.0 %) | 2 (0.9 %) | 0.8422 | 0.9450 |
| Myocardial infarction n (%) | 3 (1.7 %) | (5.0 %) | 0 (0.0 %) | 0.9954 | 0.9924 |
| New onset atrial fibrillation n (%) | 42 (23.3 %) | (19.2 %) | 52 (24.2 %) | 0.8429 | 0.2380 |
| New onset pacemaker n (%) | 7 (5.5 %) | (7.8 %) | 13 (11.2 %) | 0.1135 | 0.3779 |
| Bleeding ≥2 RBC units n (%) | 96 (88.9 %) | (91.6 %) | 52 (86.7 %) | 0.6704 | 0.3127 |
| Vascular complication n (%) | 0 (0.0 %) | (0.0 %) | 18 (8.1 %) | 0.9890 | 0.9899 |
| Antiplatelet drug n (%) | 104 (57.8 %) | (50.5 %) | 199 (90.0 %) | <0.0001 | <0.0001 |
| Anticoagulant n (%) | 118 (65.9 %) | (62.4 %) | 110 (50.2 %) | 0.0017 | 0.0162 |
| New onset dialysis |  |  |  |  |  |
| temporary n (%) | 16 (8.8 %) | (8.9 %) | 9 (4.1 %) | 0.0530 | 0.0524 |
| chronic n (%) | 2 (1.1 %) | (0.2 %) | 1 (0.5 %) | 0.4620 | 0.7243 |
| Post-OP stay in ICU (days), mean (se) | 3.9 (0.4) | 4.0 (0.4) | 3.9 (0.4) | 0.9802 | 0.8753 |

CPB: cardiopulmonary bypass; HLM: heart-lung machine; ICU: intermediate care unit; RBC: red blood cell; OP: operation; SAVR: surgical aortic valve replacement; TAVI: transcatheter aortic valve replacement; TIA: transient ischemic attack

**Supplementary Table 5: Complications at 1 year after propensity score weighted adjustment. Only patients with age between 65 and 75 years are used here.**

|  | **SAVR unweighted (n=181)** | **SAVR  (n=181)** | **TAVI  (n=222)** | **p unweighted** | **p weighted** |
| --- | --- | --- | --- | --- | --- |
| **Myocardial infarction** |  |  |  | 0.5612 | 0.0367 |
| yes n/N (%) | 2 (1.4 %) | (6.3 %) | 1 (0.7 %) |  |  |
| **New Pacemaker/ICD** |  |  |  | 0.0883 | 0.4446 |
| yes n/N (%) | 12 (9.4 %) | (13.2 %) | 19 (17.0 %) |  |  |
| **Stroke** |  |  |  | 0.9823 | 0.9881 |
| yes n/N (%) | 2 (1.5 %) | (1.4 %) | 2 (1.4 %) |  |  |
| **TIA** |  |  |  | 0.4381 | 0.4110 |
| yes n/N (%) | 2 (1.5 %) | (1.4 %) | 4 (2.9 %) |  |  |
| **Bypass** |  |  |  |  |  |
| yes n/N (%) | 0 (0.0 %) | (0.0%) | 0 (0.0 %) |  |  |
| **PCI/Balloon Dilatation** |  |  |  | 0.6713 | 0.3137 |
| yes n/N (%) | 2 (1.5 %) | (0.6 %) | 3 (2.1 %) |  |  |
| **Further Hospitalization** |  |  |  | 0.0850 | 0.0141 |
| yes n/N (%) | 50 (36.2 %) | (31.6 %) | 65 (46.4 %) |  |  |
| **Further Hospitalization due to complications related to the aortic valve intervention** |  |  |  | 0.3963 | 0.0462 |
| yes n/N (%) | 9 (18.0 %) | (28.2 %) | 8 (12.3 %) |  |  |
| **Further Hospitalization due to heart or circulatory problems** |  |  |  | 0.3648 | 0.7391 |
| yes n/N (%) | 23 (46.9 %) | (41.8 %) | 25 (38.5 %) |  |  |
| **NYHA** |  |  |  |  |  |
| I n/N (%) | 49 (36.3 %) | (32.5 %) | 40 (29.0 %) | 0.1983 | 0.5416 |
| II n/N (%) | 48 (35.6 %) | (43.7 %) | 51 (37.0 %) | 0.8098 | 0.2666 |
| III n/N (%) | 33 (24.4 %) | (22.4 %) | 41 (29.7 %) | 0.3284 | 0.1850 |
| IV n/N (%) | 5 (3.7 %) | (1.3 %) | 6 (4.3 %) | 0.7869 | 0.1766 |
| **Comparison of current general health status to health status before intervention** |  |  |  |  |  |
| better n/N (%) | 81 (59.6 %) | (66.5 %) | 92 (66.2 %) | 0.2557 | 0.9525 |
| same as before n/N (%) | 34 (25.0 %) | (15.9 %) | 28 (20.1 %) | 0.3360 | 0.3706 |
| worse n/N (%) | 21 (15.4 %) | (17.6 %) | 19 (13.7 %) | 0.6770 | 0.3818 |
| **Patient satisfaction with the intervention** |  |  |  |  |  |
| very good n/N (%) | 59 (43.7 %) | (40.7 %) | 71 (51.4 %) | 0.2006 | 0.0818 |
| good n/N (%) | 58 (43.0 %) | (43.1 %) | 42 (30.4 %) | 0.0324 | 0.0348 |
| satisfactory n/N (%) | 12 (8.9 %) | (12.6 %) | 16 (11.6 %) | 0.4625 | 0.7947 |
| not satisfactory n/N (%) | 6 (4.4 %) | (3.6 %) | 9 (6.5 %) | 0.4540 | 0.2947 |

ICD: implantable cardioverter defibrillator; NYHA: New York Heart Association; PCI: percutaneous coronary intervention; SAVR: surgical aortic valve replacement; TAVI: transcatheter aortic valve replacement; TIA: transient ischemic attack
